# Inter-rater agreement in the assessment of abnormal chest X-ray findings for tuberculosis between two Asian countries

**DOI:** 10.1186/1471-2334-12-31

**Published:** 2012-02-01

**Authors:** Shinsaku Sakurada, Nguyen TL Hang, Naoki Ishizuka, Emiko Toyota, Le D Hung, Pham T Chuc, Luu T Lien, Pham H Thuong, Pham TN Bich, Naoto Keicho, Nobuyuki Kobayashi

**Affiliations:** 1 National Center for Global Health and Medicine, Tokyo, Japan; 2 NCGM-BMH Medical Collaboration Center, Hanoi, Viet Nam; 3 NHO Tokyo Hospital, Tokyo, Japan; 4 Hanoi Lung Hospital, Hanoi, Viet Nam

## Abstract

**Background:**

Inter-rater agreement in the interpretation of chest X-ray (CXR) films is crucial for clinical and epidemiological studies of tuberculosis. We compared the readings of CXR films used for a survey of tuberculosis between raters from two Asian countries.

**Methods:**

Of the 11,624 people enrolled in a prevalence survey in Hanoi, Viet Nam, in 2003, we studied 258 individuals whose CXR films did not exclude the possibility of active tuberculosis. Follow-up films obtained from accessible individuals in 2006 were also analyzed. Two Japanese and two Vietnamese raters read the CXR films based on a coding system proposed by Den Boon et al. and another system newly developed in this study. Inter-rater agreement was evaluated by kappa statistics. Marginal homogeneity was evaluated by the generalized estimating equation (GEE).

**Results:**

CXR findings suspected of tuberculosis differed between the four raters. The frequencies of infiltrates and fibrosis/scarring detected on the films significantly differed between the raters from the two countries (*P *< 0.0001 and *P *= 0.0082, respectively, by GEE). The definition of findings such as primary cavity, used in the coding systems also affected the degree of agreement.

**Conclusions:**

CXR findings were inconsistent between the raters with different backgrounds. High inter-rater agreement is a component necessary for an optimal CXR coding system, particularly in international studies. An analysis of reading results and a thorough discussion to achieve a consensus would be necessary to achieve further consistency and high quality of reading.

## Background

Despite its several disadvantages, chest radiography remains an important supporting tool in tuberculosis (TB) surveys and clinical management of active disease [1-3]. Chest X-ray (CXR) findings should be carefully assessed because of its potential problems such as low specificity and insufficient reproducibility [4].

In this context, reading methods that are less influenced by raters are required and several CXR coding systems have been proposed [5-7]. In general, complex interpretation codes hamper intra- and inter-rater agreement and simple codes are preferred [6,7], because reproducible and validated coding system may be useful in monitoring disease in clinical and epidemiological studies [8,9].

Previous studies suggest that variability in CXR interpretation among raters is attributed to subjective reading accompanied by insufficient experience or different professional background of the raters [7,10-12]. However, the relationship between agreement levels and relevant factors that may cause disagreement, particularly influence of medical background including different national origins has not been characterized.

In the present study, Vietnamese and Japanese raters studied the readings of suspected TB lesions on CXR films taken during a survey of TB prevalence in Hanoi, Viet Nam [3]. The follow-up films were also compared with the initial films. As analytical tools, two different types of coding systems were used: One was previously reported by another group [5] and the other was newly developed in this study. The aim of the study was to highlight inter-rater agreement between raters with different medical backgrounds. We also attempted to characterize the optimal codes or coding systems used in international studies for a simple and objective evaluation of CXR findings suspected of TB.

## Methods

### Ethics approval

This study was approved by the ethics committees of the Ministry of Health, Viet Nam and the National Center for Global Health and Medicine (formerly, International Medical Center of Japan). Written informed consent was obtained from each participant prior to the investigations, including the prevalence survey and the follow-up study.

### Study population

A population-based TB prevalence survey of 11,624 people aged 15 and over was conducted in Hanoi in 2003 as reported previously [3]. Briefly, subjects suspected of having active TB based on CXR or on symptoms underwent sputum smear microscopy and/or mycobacterial culture. Details of HIV status were not obtained from the study subjects. According to the report of World Health Organization during this period, estimated prevalence of HIV co-infection in new TB patients aged 15-49 was relatively low (2.8%) in Viet Nam [13].

Barring 317 individuals, active TB was radiographically excluded for the rest. Of these 317 individuals, 22 (6.9%) were diagnosed by bacteriological methods, including sputum culture [3]. In 2004, individuals who presented with radiographic findings during the initial survey were advised to undergo sputum smear and culture tests following the World Health Organization recommendation [14,15]. In the 2006 follow-up, in which the same group of individuals was recalled for plain chest radiographic examination (AGFA X-ray film, Beijing, China; Shimadzu UD 150L-30V, Kyoto, Japan) and sputum test, including direct smear and culture. Using a questionnaire, we collected information regarding individual history, additional examinations performed, and treatment for TB undergone after the initial survey. Demographic information (including addresses) collected during the prevalence survey was used to trace the target group in the follow-up period.

The CXR films analyzed in this study were those in which active TB had not been radiographically excluded during the prevalence survey and were those taken during the follow-up in 2006. In total, 258 of the 317 films in the prevalence survey and 93 follow-up films were available at the time of analysis in this study. The rest of TB-suspected films in the prevalence survey were missing.

### CXR coding systems and reading of films

**Figure 1:**
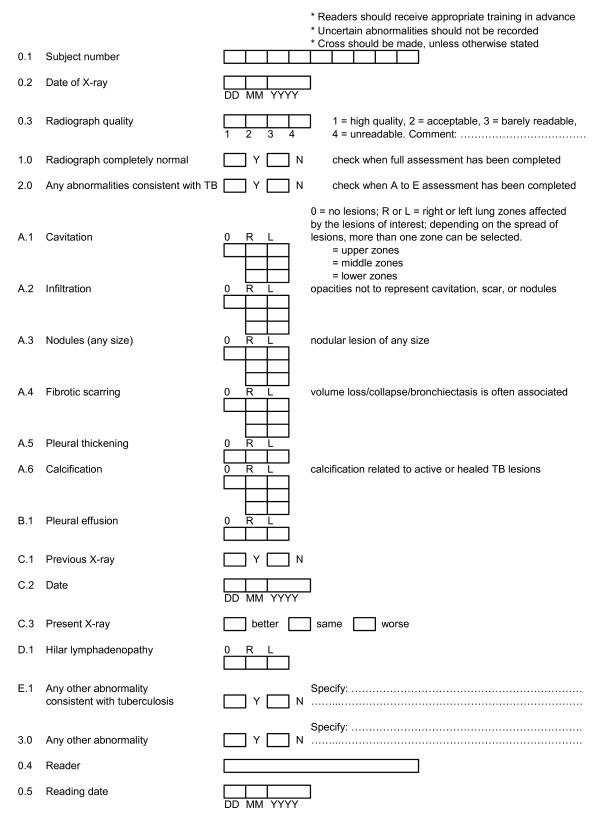
**Chest X-ray coding: JVCS**. JVCS = Japan-Vietnam chest X-ray coding system; DD = date in two digits; MM = month in two digits; YYYY = year in four digits; Y = yes; N = no; R = right; L = left.

Two coding systems were used to classify the CXR findings. The chest radiograph reading and recording system (CRRS) was developed in 2005 to detect TB and other forms of lung disease [5]. Profusion score and details of abnormalities unrelated to TB were omitted. All the other coding items of this system were retained. A Japan-Vietnam CXR coding system (JVCS) (Figure 1) consisting of rather simple codes was also used: We newly developed this system, considering a registration form used in a public payment system for TB treatment expenses in Japan and reading practice in Viet Nam. CRRS classifies parenchymal abnormalities as primary or secondary lesions depending on the significance of the lesion. In contrast, JVCS does not consider the significance of the lesion, though it records pleural effusion and thickening separately. Additionally, CRRS classifies nodules based on their size and calcification, whereas JVCS separately records nodules and calcification.

Two Japanese pulmonary physicians (E.T. and N.K.) and two Vietnamese radiologists (L.D.H. and P.T.C.) read the CXR films. These readers were different from those who read the CXR films during the initial survey. All CXR films were first read using CRRS. After the completion of readings by CRRS, CXR films were read using JVCS without the results of CRRS being made known to the readers. Each reader was also blinded to the others' readings and clinical information. Instruction and training regarding the two coding systems were given prior to the actual reading. The four raters were asked to reach a consensus while assessing 10 standard films from Japan and another 10 films from Viet Nam.

### Statistical analysis

We adopted a double entry system of data entry. JMP version 7.0.1 (SAS Institute Inc., Cary, NC, USA) and SAS version 9.1 (SAS Institute Inc.) were used for analysis. Kappa statistics were used to investigate inter-rater agreement on the presence or absence of lesions of interest. We adopted the following guidelines for interpretation of kappa coefficients: < 0, poor agreement; 0-0.20, slight; 0.21-0.40, fair; 0.41-0.60, moderate; 0.61-0.80, good; and 0.81-1.00, very good [16-18]. Weighted kappa was used to assess inter-rater agreement on variables with more than two categories. McNemar's test or its extension, Bowker's test of symmetry, was used to investigate the symmetry of disagreement between two raters, which tests whether the frequency of an abnormality detected by one rater is significantly different from that by another rater. The generalized estimation equation (GEE) was also used to test the similarities in frequencies of positive findings between groups of raters (marginal homogeneity). No symmetry or non-marginal homogeneity was considered to be significant when *P *< 0.05.

## Results

### Follow-up after TB prevalence survey

In 2004, one year after the prevalence survey, 204 (64.4%) of the 317 individuals who presented with radiographic findings of suspected TB underwent a sputum smear test, one of whom tested positive. The initial CXR film of this case showed infiltrates, fibrosis/scarring, and calcification. The follow-up radiograph in 2006 showed improvement after treatment.

**Figure 2:**
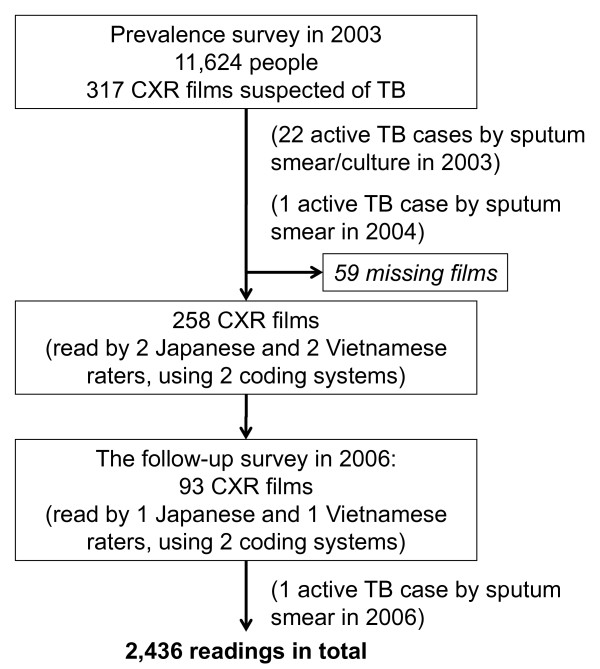
**Chest X-ray films for reading**. TB = tuberculosis; CXR = chest X-ray.

In the follow-up in 2006, 93 individuals were checked, one of whom was diagnosed by smear and culture as TB positive (Figure 2). Besides calcification, which was seen in the initial CXR film, infiltrates were present in the follow-up film. All raters evaluated this case as "worse" based on the radiographic findings.

In total, five individuals were reported to have active TB during the 3-year follow-up period. Two were diagnosed bacteriologically and three were diagnosed based on self-reported TB episodes. All the films were randomly mixed in the study set.

### Inter-rater agreement on CXR findings

Using the two coding systems, four raters assessed the 258 films taken during the 2003 prevalence survey; two raters assessed the 93 films taken in the 2006 follow-up. A total of 2,436 readings were conducted (Figure 2).

Agreement levels regarding overall parenchymal abnormalities assessed by CRRS varied. Their kappa values were interpreted as fair to good, ranging from 0.24 to 0.63, from the following six comparisons: a comparison between the two Japanese raters (JP-JP); four comparisons between Japanese and Vietnamese raters (JP-VN (1) to (4)); and a comparison between the two Vietnamese raters (VN-VN) (Table 1). Agreement levels regarding calcification also varied. They were considered as fair to good with JVCS and slight to fair with CRRS. Kappa values for pleural effusion with JVCS were interpreted as moderate to good, ranging from 0.54 to 0.77, indicating high level of agreement irrespective of country or rater.

**Table 1:** Inter-rater agreement with respect to general and parenchymal findings for each coding system (n = 258)

Item	Coding	Inter-rater agreement					
	system	Kappa with 95% confidence interval and the absolute number of films (--/-+/+-/++)					
		
		JP-JP	JP-VN				VN-VN
			
			[1]	[2]	[3]	[4]	
Total number	JVCS	(245)	(246)	(245)	(245)	(244)	(255)
of tested films	CRRS	(245)	(245)	(246)	(246)	(245)	(255)
Parenchymal	JVCS	NA	NA	NA	NA	NA	NA
abnormality	CRRS	0.63 [0.51-0.75]	0.24 [0.16-0.32]	0.50 [0.38-0.62]	0.25 [0.16-0.34]	0.58 [0.46-0.70]	0.27 [0.17-0.37]
		(32/21/7/185)	(9/44/0/192)	(24/29/6/187)	(7/32/2/205)	(22/17/8/198)	(6/3/24/222)
Calcification*	JVCS	0.62 [0.49-0.75]	0.47 [0.35-0.59]	0.21 [0.12-0.30]	0.55 [0.42-0.68]	0.30 [0.21-0.39]	0.26 [0.17-0.35]
		(188/13/14/30)	(187/14/22/23)	(198/2/38/7)	(190/12/18/25)	(201/0/34/9)	(215/2/31/7)
	CRRS	0.28 [0.15-0.41]	0.35 [0.23-0.47]	0.15 [0.04-0.26]	0.36 [0.25-0.47]	0.36 [0.25-0.47]	0.17 [0.09-0.25]
		(219/10/11/5)	(206/23/6/10)	(226/4/14/2)	(208/23/5/10)	(228/2/11/4)	(219/2/30/4)
Pleural	JVCS	0.58 [0.45-0.71]	0.77 [0.64-0.90]	0.66 [0.54-0.78]	0.64 [0.51-0.77]	0.54 [0.41-0.67]	0.73 [0.61-0.85]
effusion		(222/8/5/10)	(226/5/2/13)	(222/8/3/12)	(221/6/6/12)	(217/9/7/11)	(230/6/4/15)
Pleural	JVCS	0.35 [0.23-0.47]	0.22 [0.14-0.30]	0.45 [0.33-0.57]	0.28 [0.18-0.38]	0.45 [0.32-0.58]	0.31 [0.22-0.40]
thickening		(158/39/19/29)	(88/110/2/46)	(161/36/14/34)	(84/93/6/62)	(148/28/26/42)	(87/4/95/69)
Pleural	CRRS	0.30 [0.20-0.40]	0.16 [0.09-0.23]	0.46 [0.34-0.58]	0.45 [0.33-0.57]	0.54 [0.42-0.66]	0.32 [0.22-0.42]
abnormalities		(130/73/6/36)	(83/120/3/39)	(159/44/7/36)	(76/60/10/100)	(124/12/42/67)	(81/6/91/77)
Pleural effusion/	CRRS	0.48 [0.36-0.60]	0.34 [0.22-0.46]	0.55 [0.43-0.67]	0.49 [0.36-0.62]	0.67 [0.55-0.79]	0.48 [0.36-0.60]
thickening**		(176/35/6/28)	(168/43/11/23)	(189/22/9/26)	(156/26/23/41)	(176/6/22/41)	(173/14/33/35)

* In CRRS, calcification here indicates calcified granuloma only

**In CRRS, pleural effusion and thickening are combined

*JVCS *Japan-Vietnam chest X-ray coding system, *CRRS *chest radiograph reading and recording system, *TB *tuberculosis, *NA *not applicable, *JP-JP *a comparison between the Japanese raters; *JP-VN *[1] to [4] comparisons between Japanese-Vietnamese raters, *VN-VN *a comparison between the Vietnamese raters, (--/-+/+-/++) (negative findings by both raters/positive findings only by the second rater/positive findings only by the first rater/positive findings by both raters)

Major parenchymal findings, cavity, fibrosis/scarring, infiltrates, and nodules were assessed in a similar way, as shown in Table 2. Agreement levels regarding primary and secondary cavities in CRRS were rather low (kappa values ranged from -0.02 to 0.36) except for relatively high agreement levels regarding a primary cavity between the Japanese raters (kappa = 0.60), and a secondary cavity between the Vietnamese raters (kappa = 0.43). Cavitation was, thus, mainly classified as a primary lesion by the Japanese raters and as a secondary lesion by the Vietnamese raters.

**Table 2:** Inter-rater agreement with respect to parenchymal findings for each coding system (n = 258)

Item	Coding system	Inter-rater agreement					
		Kappa with 95% confidence interval					
		
		JP-JP	JP-VN				VN-VN
			
			[1]	[2]	[3]	[4]	
Total number	JVCS	(245)	(246)	(245)	(245)	(244)	(255)
of tested films	CRRS	(245)	(245)	(246)	(246)	(245)	(255)
Cavity	JVCS	0.44 [0.32-0.56]	0.36 [0.25-0.47]	0.47 [0.34-0.64]	0.30 [0.20-0.40]	0.50 [0.38-0.62]	0.52 [0.40-0.64]
	CRRS primary *	0.60 [0.48-0.72]	0.10 [0.03-0.17]	0.28 [0.18-0.38]	0.06 [-0.02-0.14]	0.36 [0.25-0.47]	0.15 [0.04-0.26]
	CRRS secondary	-0.02 [-0.14-0.10]	0.04 [0.00-0.08]	0.06 [0.01-0.11]	0.00 [-0.05-0.05]	0.04 [-0.03-0.11]	0.43 [0.32-0.54]
Fibrosis/scar	JVCS	0.30 [0.17-0.43]	0.19 [0.07-0.31]	0.34 [0.22-0.46]	0.18 [0.05-0.31]	0.34 [0.23-0.45]	0.31 [0.20-0.42]
	CRRS primary	0.31 [0.18-0.44]	0.02 [-0.02-0.06]	0.27 [0.14-0.40]	-0.02 [-0.07-0.03]	0.15 [0.02-0.28]	0.03 [-0.01-0.07]
	CRRS secondary	0.28 [0.16-0.40]	0.20 [0.10-0.30]	0.11 [0.02-0.20]	0.14 [0.03-0.25]	0.16 [0.06-0.26]	0.22 [0.10-0.34]
Infiltrate	JVCS	0.49 [0.37-0.61]	0.30 [0.20-0.40]	0.27 [0.18-0.36]	0.22 [0.13-0.31]	0.21 [0.13-0.29]	0.57 [0.45-0.69]
	CRRS primary	0.33 [0.21-0.45]	0.24 [0.15-0.33]	0.31 [0.19-0.43]	0.15 [0.08-0.22]	0.22 [0.12-0.32]	0.41 [0.30-0.52]
	CRRS secondary	-0.05 [-0.18-0.08]	0.13 [0.03-0.23]	-0.02 [-0.12-0.08]	-0.04 [-0.14-0.06]	-0.02 [-0.12-0.08]	0.02 [-0.03-0.07]
Nodule	JVCS	0.27 [0.14-0.40]	0.11 [0.05-0.17]	0.26 [0.14-0.38]	0.09 [0.03-0.15]	0.31 [0.20-0.42]	0.19 [0.11-0.27]
	CRRS primary	0.37 [0.25-0.49]	0.13 [0.06-0.20]	0.40 [0.28-0.52]	0.09 [0.03-0.15]	0.24 [0.12-0.36]	0.21 [0.13-0.29]
	CRRS secondary	0.22 [0.10-0.34]	0.22 [0.11-0.33]	0.14 [0.02-0.26]	0.13 [0.03-0.23]	0.29 [0.16-0.42]	0.22 [0.12-0.32]

* Primary and secondary lesions are described in CRRS

*JVCS *Japan-Vietnam chest X-ray coding system, *CRRS *chest radiograph reading and recording system, *TB *tuberculosis, *NA *not applicable, *JP-JP *a comparison between the Japanese raters, *JP-VN * [1] to [4] comparisons between Japanese-Vietnamese raters, *VN-VN *a comparison between the Vietnamese raters

Although agreement levels relating to fibrosis/scarring were also low, kappa values for secondary fibrosis/scarring with CRRS revealed fair levels of agreement between raters from the same country (kappa = 0.28 [JP-JP] and 0.22 [VN-VN]), but revealed only slight agreement between raters from different countries (kappa = 0.11 to 0.20 [JP-VN]). Among all Japanese-Vietnamese pairs, the Vietnamese raters specified secondary fibrosis/scars more frequently than the Japanese raters (*P *= 0.0001 or *P *< 0.0001 by McNemar test). The frequency of positive findings of secondary fibrosis with CRRS by both Vietnamese raters was 26/255 (10.2%), whereas that by both Japanese raters was only 7/245 (2.9%) (Table not shown). The frequency of positive findings of fibrosis/scarring with JVCS by both Vietnamese raters (56/255 = 22.0%) also tended to be higher than that by both Japanese raters (42/245 = 17.1%). GEE further confirmed the significant difference in frequencies of fibrosis/scarring between raters from different countries (*P *= 0.0082).

Agreement levels regarding infiltrates between the two raters from the same country were considered as moderate (kappa = 0.49 [JP-JP] and 0.57 [VN-VN]) and as fair between two raters from different countries (kappa = 0.21 to 0.30 [JP-VN]) according to JVCS (Table 2). The Japanese raters detected infiltrates more frequently than the Vietnamese raters (*P *< 0.0001 by McNemar test) in all comparisons. The frequency of positive findings of primary infiltrates with CRRS by both Japanese raters was 68/245 (27.8%), whereas that by both Vietnamese raters was only 22/255 (8.6%) (Table not shown). The frequency of positive findings of infiltrates with JVCS by both Japanese raters (119/245 = 48.6%) also tended to be higher than that by both Vietnamese raters (46/255 = 18.0%). The different frequencies of positive infiltrate readings between the raters from the two countries were also confirmed by using GEE (*P *< 0.0001).

The levels of inter-rater agreement were considered slight to fair for nodules, irrespective of the raters' home country or the coding system used.

**Figure 3:**
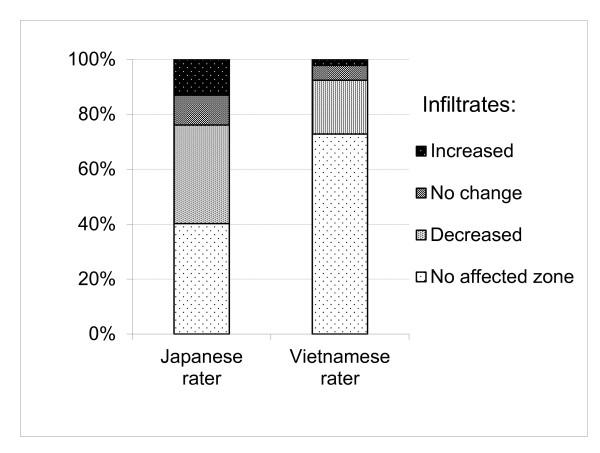
**Infiltrates on chest X-rays after a 3-year interval**. Evaluation of infiltrates on chest X-ray films between 2003 and 2006 using JVCS codes shows changes in the number of affected areas (upper, middle, and lower zones of each side of the lung). "No affected zone" indicates that the rater did not indicate the presence of infiltrates in either film. The Japanese rater detected infiltrates more commonly than did the Vietnamese rater (*P *< 0.0001), which corresponds with the results of a greater proportion of "increased" and a smaller proportion of "no detected zone" readings after 3 years. JVCS = Japan-Vietnam chest X-ray coding system

An overall assessment of CXR changes after 3 years was conducted by one of the two raters from each country. Agreement was moderate for both coding systems (weighted kappa = 0.47 and 0.40). The Japanese rater indicated deterioration more frequently than the Vietnamese rater (Table 3); this difference was considered highly significant for both JVCS and CRRS by the symmetry test (*P *= 0.0002 and 0.0008, respectively, by Bowker's test). When assessing changes in specific findings after 3 years, the Japanese rater detected infiltrates more frequently than the Vietnamese rater (*P *< 0.0001; Figure 3). Among 55 cases of infiltrates, 12 (22%) were assessed as "further spread" by the Japanese rater while 2 (8%) out of 25 cases of infiltrates were assessed as "further spread" by the Vietnamese rater (data not shown).

**Table 3:** Overall assessment of radiographic findings after 3 years

JVCS					CRRS				
**JP**	**VN**			**Total**	**JP**	**VN**			**Total**
					
	**Better**	**Same**	**Worse**			**Better**	**Same**	**Worse**	

Better	23	6	0	29	Better	28	6	0	34
Same	18	21	0	39	Same	16	18	1	35
Worse	7	7	7	21	Worse	4	10	5	19
Total	48	34	7	89	Total	48	34	6	88
Weighted kappa = 0.40 [0.22-0.57]					Weighted kappa = 0.47 [0.31-0.63]				

*JVCS *Japan-Vietnam chest X-ray coding system, *CRRS *chest radiograph reading and recording system, *JP *Japanese rater, *VN *Vietnamese rater

## Discussion

Our study confirmed that the readings of CXR findings of suspected TB vary significantly among the raters. Differences in the backgrounds of the raters and different coding systems were considered potential factors affecting the levels of agreement. We found the following two patterns of marked tendency toward inconsistency in the CXR findings: 1) disagreement presumably attributed to the raters' home country and typically observed for infiltrates and secondary fibrosis/scarring and 2) disagreement observed for nodules, irrespective of the rater background. Through discussions conducted with the four raters after the trial, we identified some possible causes of this disagreement, though pre-existing problems were not disclosed when the standard films were checked prior to commencement of the study.

First, it is likely that this disagreement was partly caused by differences between countries regarding the definition of pulmonary lesions. For example, the Vietnamese raters limited the definition of infiltrates to relatively homogenous opacities greater than 10 mm in size, whereas the Japanese raters also included groups of smaller-sized scattered lesions with unclear margins in this classification. As a result, positive findings of infiltrates were more frequently reported by the Japanese raters.

Second, spontaneously cured mild TB resulting in parenchymal fibrosis or scarring, which is commonly seen in countries with high prevalence of TB, is a probable reason for the more frequent detection of these lesions by the Vietnamese raters. In addition, CT scans are compared with plain CXRs more commonly in Japan than in Viet Nam. This practice in TB diagnosis and management might affect the interpretations of the Japanese raters.

Disagreement between the raters from the two Asian countries could be attributed to many background factors, including the medical educational systems and on-the-job training imparted after graduation. In Japan, plain CXR films are read predominantly by clinicians, while in Viet Nam, radiologists also perform this role. Such differences are likely to affect the reading and should be taken into consideration in international studies. Even within a single country, inter-rater agreement depends on the experience of the raters [7,10,12] and is relatively low between raters in different centers [10].

The tested coding systems had both advantages and disadvantages in the context of our study. With CRRS, parenchymal abnormalities are classified into primary and secondary lesions, and it is not easy for raters to differentiate between the two. The Japanese raters emphasized on cavitation and presence of infiltrates as primary lesions of active TB, but the Vietnamese raters objectively judged the primary lesions on the basis of the size of lesions and proportion of the lung involved.

Although fairly reproducible, a disadvantage of JVCS is that it cannot provide any information regarding the significance of active lesions. Thus, CRRS is more informative. Activity, however, is a subjective term and the reproducibility of this description apparently worsens when included in a coding system. This implies the limitations of the plain CXR as a classic imaging tool. It may be assumed that defining necessary medical terms carefully through training and in-depth discussion prior to actual reading would minimize misunderstandings, even with a detailed coding system. However, this was not effective in our study, possibly because of language barriers, different medical backgrounds, and insufficient recognition of the problems. Collectively, our results support the concept of reproducibility of a simplified coding system [6,7,19], which may be critical when a system is shared by raters from different countries, such as even Asian countries.

On comparing CXR findings 3 years after the prevalence survey, Japanese raters detected deterioration in more cases than Vietnamese raters. The fact that the Japanese raters more frequently detected infiltrates may partly explain this discrepancy, because infiltrates generally signify active lesions, though unknown factors may also have affected their readings. This should be considered when CXRs are used for follow-up because the radiological appearance of lesions will not provide sufficient information for monitoring TB unless patient history and bacteriological examination are combined [8,10,19].

Our study has several limitations. First, caution should be exercised when extrapolating the results to describe the way CXRs are generally read in the two Asian countries. Although different medical backgrounds in the countries were obvious after reviewing and discussing the results, the raters' qualifications should also be considered. Second, in the present study, the overall sensitivity and specificity of CXR-based diagnosis of tuberculosis were not determined because the number of active TB cases detected in our cohort study was rather small (< 10%) and because these parameters would be influenced more by individual raters' skills and experiences than by the coding system used. Third, the coverage rate of the radiographic follow-up study after 3 years was not high, one of the reasons being the rapid speed of urbanization and an increasingly mobile population in Hanoi, which caused difficulties when tracing particular individuals. Nevertheless, our findings present an important point to be considered in international studies of TB using a CXR coding system.

## Conclusions

In our study, CXR findings of suspected TB were inconsistent between raters with different backgrounds, presumably because of differences in medical practice and education between the two countries. Although each coding system has its advantages and disadvantages, a simplified classification system is suitable for maintaining sufficient agreement between raters from different countries. To improve the quality of future international collaborative studies, harmony could be obtained between raters of different nationalities by thorough discussion regarding the possible causes of disagreement in CXR readings, using standard films and descriptions of major findings.

## Competing interests

The authors declare that they have no competing interests.

## Authors' contributions

SS and NTLH participated in supervising the on-site implementation of the study, drafting the paper, and substantially revising it. ET, LDH, PTC, and NKo read the chest X-ray films. LTL and PHT participated in the conception, design, and supervision of the study. PTNB participated in on-site implementation of the study. NI supervised and performed statistical analysis. NKe participated in the conception and design of the study, analysis and interpretation of data, drafting of the paper, and substantially revising it. All authors read and approved the final manuscript.

## Pre-publication history

The pre-publication history for this paper can be accessed here:

http://www.biomedcentral.com/1471-2334/12/31/prepub

## References

[B1] Hopewell PC Pai M Maher D Uplekar M Raviglione MC International standards for tuberculosis care Lancet Infect Dis 2006 6 710 725 10.1016/S1473-3099(06)70628-4 17067920

[B2] Golub JE Mohan CI Comstock GW Chaisson RE Active case finding of tuberculosis: historical perspective and future prospects Int J Tuberc Lung Dis 2005 9 1183 1203 16333924 PMC4472641

[B3] Horie T Lien LT Tuan LA A survey of tuberculosis prevalence in Hanoi, Vietnam Int J Tuberc Lung Dis 2007 11 562 566 17439682

[B4] Koppaka R Bock N How reliable is chest radiography? Toman's Tuberculosis Case detection, treatment, and monitoring--questions and answers 2004 2 Geneva: World Health Organization 51 6022299450

[B5] Den Boon S Bateman ED Enarson DA Development and evaluation of a new chest radiograph reading and recording system for epidemiological surveys of tuberculosis and lung disease Int J Tuberc Lung Dis 2005 9 1088 1096 16229219

[B6] Graham S Das GK Hidvegi RJ Chest radiograph abnormalities associated with tuberculosis: reproducibility and yield of active cases Int J Tuberc Lung Dis 2002 6 137 142 11931412

[B7] Zellweger JP Heinzer R Touray M Vidondo B Altpeter E Intra-observer and overall agreement in the radiological assessment of tuberculosis Int J Tuberc Lung Dis 2006 10 1123 1126 17044205

[B8] Linh NN Marks GB Crawford AB Radiographic predictors of subsequent reactivation of tuberculosis Int J Tuberc Lung Dis 2007 11 136 1142 17945072

[B9] Ralph AP Ardian M Wiguna A A simple, valid, numerical score for grading chest x-ray severity in adult smear-positive pulmonary tuberculosis Thorax 2010 65 863 869 10.1136/thx.2010.136242 20861290

[B10] Balabanova Y Coker R Fedorin I Variability in interpretation of chest radiographs among Russian clinicians and implications for screening programmes: observational study BMJ 2005 331 379 382 10.1136/bmj.331.7513.379 16096305 PMC1184248

[B11] Brealey S Westwood M Are you reading what we are reading? The effect of who interprets medical images on estimates of diagnostic test accuracy in systematic reviews Br J Radiol 2007 80 674 677 10.1259/bjr/83042364 17762057

[B12] Abubakar I Story A Lipman M Diagnostic accuracy of digital chest radiography for pulmonary tuberculosis in a UK urban population Eur Respir J 2010 35 689 692 10.1183/09031936.00136609 20190334

[B13] Global tuberculosis control--surveillance, planning, financing. WHO Report 2005 http://www.who.int/tb/publications/global_report/2005/en/index.html WHO/HTM/TB/2005.349

[B14] Kantor IN Kim SJ Frieden T Laboratory Service in Tuberculosis Control Part II: Microscopy WHO/TB/98.258 1998

[B15] Kantor IN Kim SJ Frieden T Laboratory Service in Tuberculosis Control Part III: Culture 1998 WHO/TB/98.258

[B16] Landis JR Koch GG The measurement of observer agreement for categorical data. *Biometrics* 1977 33 159 174 843571

[B17] Kundel HL Polansky M Measurement of observer agreement Radiology 2003 228 303 308 10.1148/radiol.2282011860 12819342

[B18] Taplin SH Rutter CM Elmore JG Seger D White D Brenner RJ Accuracy of screening mammography using single versus independent double interpretation AJR Am J Roentgenol 2000 174 1257 1262 10789773 10.2214/ajr.174.5.1741257

[B19] Van Cleeff MR Kivihya-Ndugga LE Meme H Odhiambo JA Klatser PR The role and performance of chest X-ray for the diagnosis of tuberculosis: a cost-effectiveness analysis in Nairobi, Kenya BMC Infect Dis 2005 5 111 10.1186/1471-2334-5-111 16343340 PMC1326228

